# A muscle stem cell for every muscle: variability of satellite cell biology among different muscle groups

**DOI:** 10.3389/fnagi.2015.00190

**Published:** 2015-10-07

**Authors:** Matthew E. Randolph, Grace K. Pavlath

**Affiliations:** Department of Pharmacology, Emory University, Atlanta, GAUSA

**Keywords:** satellite cell, muscle, muscular dystrophy, muscle stem cell, hypaxial, epaxial, diaphragm, craniofacial

## Abstract

The human body contains approximately 640 individual skeletal muscles. Despite the fact that all of these muscles are composed of striated muscle tissue, the biology of these muscles and their associated muscle stem cell populations are quite diverse. Skeletal muscles are affected differentially by various muscular dystrophies (MDs), such that certain genetic mutations specifically alter muscle function in only a subset of muscles. Additionally, defective muscle stem cells have been implicated in the pathology of some MDs. The biology of muscle stem cells varies depending on the muscles with which they are associated. Here we review the biology of skeletal muscle stem cell populations of eight different muscle groups. Understanding the biological variation of skeletal muscles and their resident stem cells could provide valuable insight into mechanisms underlying the susceptibility of certain muscles to myopathic disease.

## Introduction

Skeletal muscle is a highly organized tissue that comprises up to 40% of a human’s body mass and is required for essential functions such as metabolism, locomotion and breathing (Janssen et al., 2000; MacIntosh et al., 2006). The human body contains approximately 640 unique skeletal muscles, each having distinct functions in human physiology. Multiple factors contribute to skeletal muscle diversity including embryologic origin, myogenic regulatory pathways, and functional/metabolic requirements. Muscle heterogeneity is further underscored by the variable sensitivity of specific subsets of skeletal muscles to numerous distinct genetic mutations that give rise to muscular dystrophies (MD; Hoffman et al., 1987; Monaco et al., 1988; Bione et al., 1994; Brais et al., 1998; Bonne et al., 1999; Nonaka, 1999; Emery, 2002; Robinson et al., 2005; Bonnemann et al., 2014; Vieira et al., 2014). Adult muscle stem cells, called satellite cells, have been implicated in the pathology of some MDs (**Table 1**) and may contribute to the variable muscle sensitivity observed in some dystrophies.

**Table 1:** Muscular dystrophies (MDs) in which deficits in satellite cell function have been implicated in disease pathology.

Muscular dystrophy	Affected muscles	Mutant gene(s)	Affected protein(s)	Altered SC function	Reference
Duchenne	shoulder, upper limb, diaphragm, and calf	*DMD*	Dystrophin	Replicative exhaustion; Proliferation; Postnatal myofiber hypotrophy	Blau et al., 1983; Webster and Blau, 1990; Sacco et al., 2010; Jiang et al., 2014; Duddy et al., 2015
Limb girdle:					
LGMD1B	upper limb, shoulder,	*LMNA*	Lamin A/C	Differentiation	Frock et al., 2006
LGMD2A	chest, hip, and upper leg	*CAPN3*	Calpain 3	Proliferation to differentiation transition	Rosales et al., 2013
LGMD2C		*SGCG*	γ-Sarcoglycan	Decreased number; Replicative impairment?	Higuchi et al., 1999


LGMD2D		*SGCA*	α-Sarcoglycan		
LGMD2E		*SGCB*	β-Sarcoglycan		
LGMD2F		*SGCD*	δ-Sarcoglycan		
LGMD2H		*TRIM32*	tripartite motif-containing 32	Replicative senescence	Kudryashova et al., 2012; Mokhonova et al., 2015
LGMD2O		*POMGNT1*	Protein O-mannose beta-1,2-*N*-acetylglucos-aminyltransferase	Proliferation	Miyagoe-Suzuki et al., 2009
Emery–Dreifuss:					
EGMD2 EGMD3	shoulder, upper limb, and calf	*LMNA*	Lamin A/C	Differentiation	Favreau et al., 2004; Frock et al., 2006
EGMD1		*EMD*	Emerin	Proliferation; Differentiation	Frock et al., 2006; Meinke et al., 2015
Facioscapulo-humeral					
FSHD1	facial, shoulder, upper arm, foot, and pelvic-girdle	*Chrom.4q35 D4Z4* contraction	*DUX4*	Differentiation; Myoblast toxicity	Kowaljow et al., 2007; Bosnakovski et al., 2008a,b
			DUX4c	Proliferation; Differentiation	Bosnakovski et al., 2008a; Ansseau et al., 2009
			FSHD region gene 1	Proliferation; Differentiation; Fusion	Chen et al., 2011; Feeney et al., 2015
Myotonic dystrophy:					
DM1	eyelid, face, neck, lower arms/legs, diaphragm, intercostals	*DMPK*	Dystophia myotonic protein kinase	Decreased satellite cell numbers Proliferation Replicative senescence	Beffy et al., 2010 Thornell et al., 2009 Bigot et al., 2009
DM2	eyelid, face, neck, upper arms/legs, diaphragm, intercostals	*ZNF9*	Zinc finger protein 9	Differentiation; Replicative senescence	Malatesta et al., 2011; Beaulieu et al., 2012; Renna et al., 2014
Oculopharyngeal	upper eyelid, EOM, pharynx, tongue, upper arms/legs	*PABPN1*	Poly adenosine binding protein-nuclear one	Proliferation Differentiation	Périé et al., 2006 Apponi et al., 2010
Congenital MD:					
Bethlem Ullrich	upper and lower arms/legs, neck, lumbar paravertebral, intercostals, thigh, gluteus maximus	*COL6A1* *COL6A2* *COL6A3*	Collagen 6A	Self-renewal	Urciuolo et al., 2013; Gattazzo et al., 2014
Rigid spine syndrome related to *SEPN1*	paravertebral, intercostals, thigh, gluteus maximus	*SEPN1*	Selenoprotein N	Decreased satellite cell numbers; Proliferation; Exhaustion of satellite cell pool	Castets et al., 2011

Skeletal muscles are composed of myofibers, large syncytial cells containing hundreds of post-mitotic myonuclei. Juxtaposed between the basal lamina and the myofiber cell membrane, satellite cells reside at the periphery of skeletal myofibers (Mauro, 1961). Recent studies have demonstrated that satellite cells expressing *paired box protein 7 (Pax7)* are the primary myogenic cell required for muscle regeneration (Lepper et al., 2011; Murphy et al., 2011; Sambasivan et al., 2011). The majority of knowledge concerning satellite cell biology arises from studies examining somite-derived muscles such as the quadriceps, gastrocnemius, tibialis anterior (TA), soleus, extensor digitorum longus, plantaris, biceps, and deltoid muscles, which collectively represent less than 2% of all skeletal muscles. Intriguingly, satellite cells present in other muscle groups, including trunk, diaphragm, larynx, tongue, extraocular, masseter, and pharynx, deviate from the canonical biology of their limb counterparts. Here, we address the muscle-specific variability of satellite cell biology and postulate how this variability could contribute to muscle-specific sensitivities found in MDs.

## Limb Muscle Satellite Cells: Establishing the Canon

**FIGURE 1:**
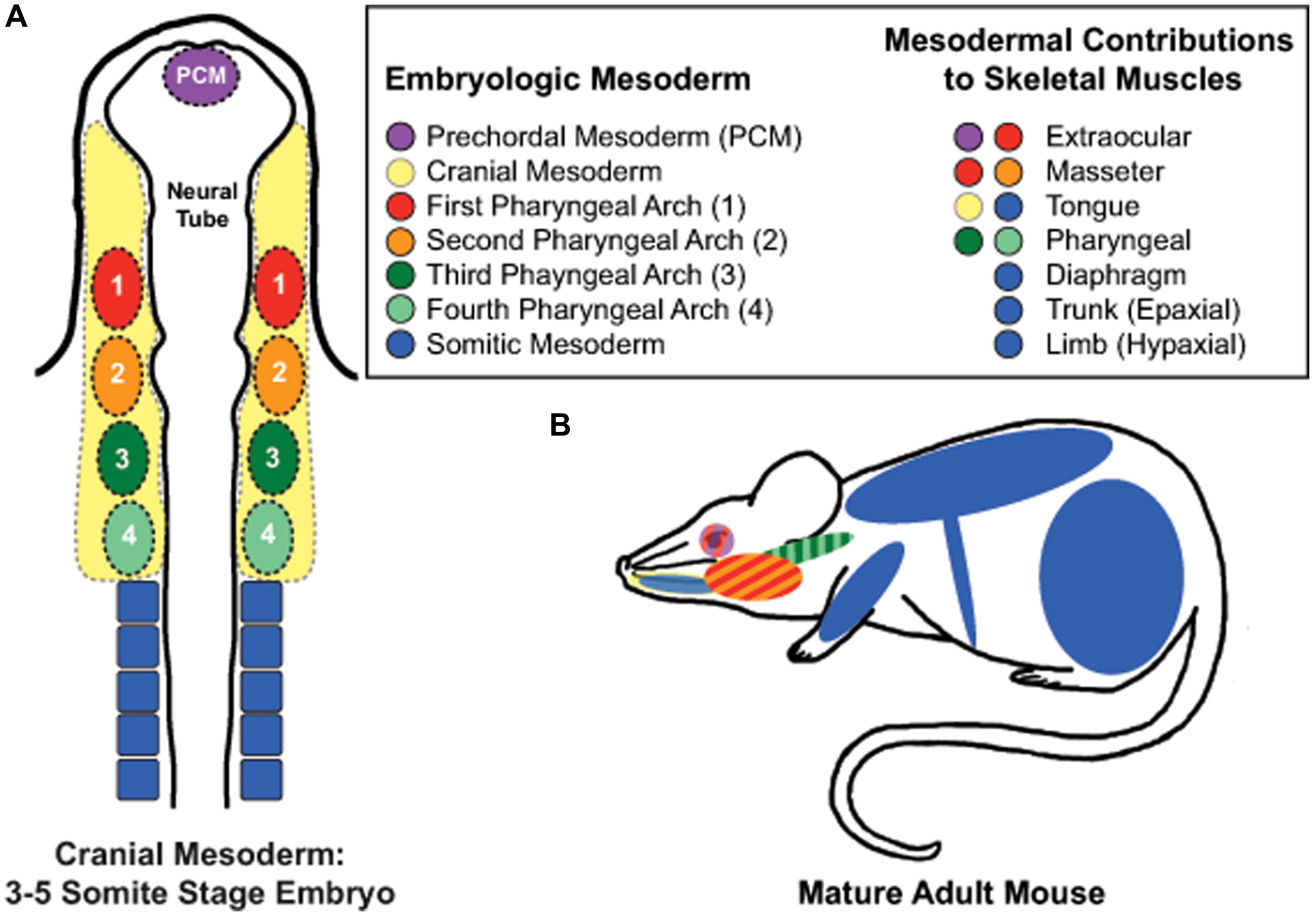
**Embryonic mesodermal contributions to adult skeletal muscles. (A)** Schematic of mesodermal origins in a 3–5 somite stage mouse embryo. **(B)** Skeletal muscles of the trunk, limb, diaphgram, and tongue arise from somitic mesoderm. In contrast, the extraocular muscles (EOMs) arise from prechordal mesoderm and cranial paraxial mesoderm of the first pharyngeal arch; the masseter muscle from the first and second pharyngeal arches of the cranial paraxial mesoderm, and the pharynx from the third and fourth pharyngeal arches of the caudal paraxial mesoderm. Tongue muscles arise from both somitic and cranial mesoderm while developing within the niche of the cranial mesenchyme, which is supplied by all four pharyngeal arches.

Skeletal muscles of rodent hindlimbs are commonly used to study satellite cells as these muscles are easy to identify, dissect, collect, and manipulate experimentally. The skeletal muscles of the limbs and abdomen arise from somitic mesoderm and are referred to as hypaxial muscles (**Figure 1**). They arise developmentally from the ventrolateral dermomyotome of the segmented paraxial mesoderm. *In vivo* and *in vitro* studies examining limb muscles provide fundamental insights into the mechanisms and regulatory pathways involved with skeletal muscle regeneration, muscle growth, and satellite cell biology.

**FIGURE 2:**
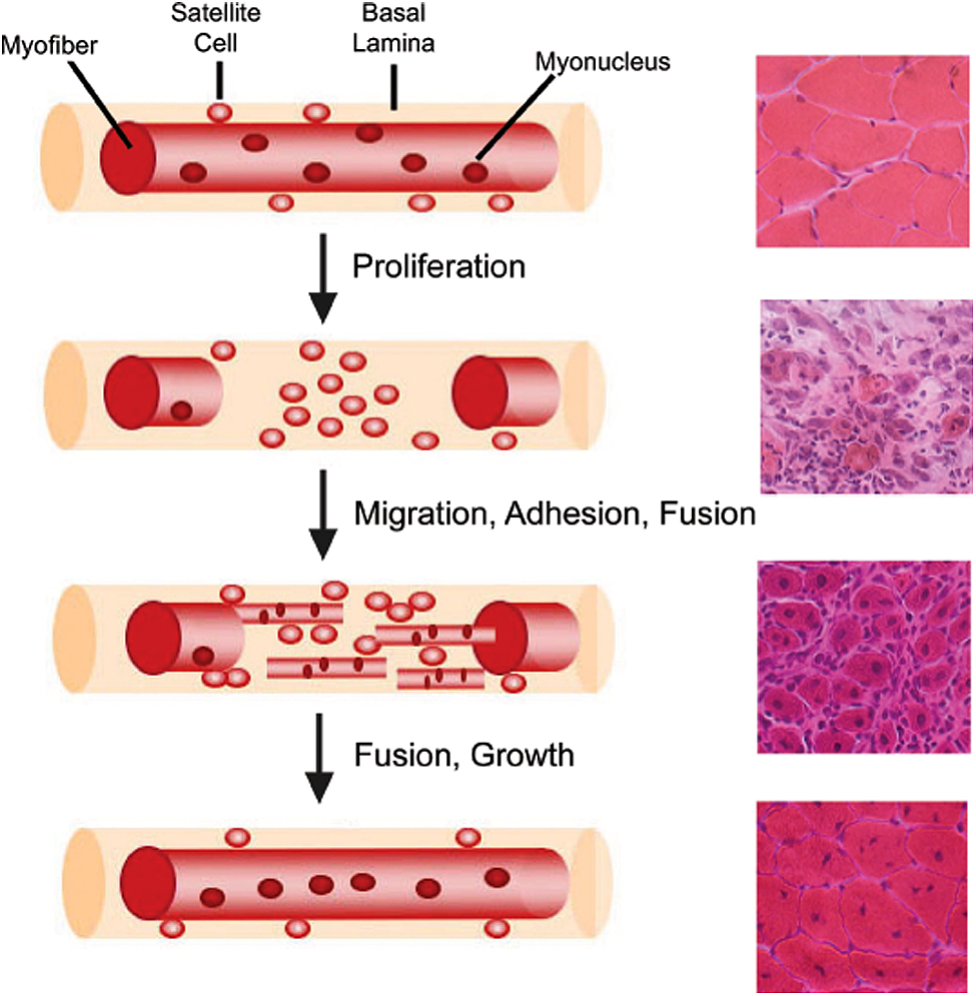
**Myofiber structure and cellular progression of myogenesis.** Myofibers are surrounded by a basal lamina, underneath which lie satellite cells in close apposition to the myofiber. With injury, satellite cells proliferate and give rise to myoblasts, which differentiate, migrate, adhere, and fuse with one another to form multiple myotubes within the basal lamina scaffold. Myoblasts/myotubes fuse with the stumps of the surviving myofiber and myotubes also fuse with each other to repair the injured myofiber. Regenerated myofibers are identifiable by the presence of centrally located nuclei. Representative hematoxylin and eosin stained muscle cross-sections from chemically injured murine muscles are provided for each stage of muscle regeneration to illustrate the differential tissue morphology.

Muscle regeneration is a robust and complex cellular process that restores injured muscle to a state that is morphologically and functionally similar to that of uninjured muscle (**Figure 2**; Abmayr and Pavlath, 2012). Regeneration of skeletal muscle occurs in two distinct phases: a degenerative phase and a regenerative phase (Rai et al., 2014). The main characteristics of the degenerative phase involve myofiber sarcolemmal damage or myofiber necrosis, followed by an influx of mononucleated inflammatory cells and an increase in fibroblasts (Mathew et al., 2011; Murphy et al., 2011; Rai et al., 2014). Factors released from damaged myofibers initiate an inflammatory response that recruits neutrophils, macrophages, and activates fibro/adipogenic progenitors to facilitate the removal of cellular debris and regulate muscle repair (McLennan, 1996; Lescaudron et al., 1999; Joe et al., 2010; Uezumi et al., 2010; Pallafacchina et al., 2013). The basal lamina remains intact acting as a scaffold for the next phase, muscle regeneration (Schmalbruch, 1976). Several molecular signals, such as growth factors, chemokines, and cytokines, are released which activate satellite cells both locally and systemically within the first 24–48 h following injury (Chang and Rudnicki, 2014; Rodgers et al., 2014). Myoblasts then terminally differentiate becoming post-mitotic myocytes, which then fuse with other myocytes or myofibers to regenerate or repair damaged myofibers. Thereby, new myonuclei are added to damaged or nascent myofibers (Abmayr and Pavlath, 2012). A subset of myogenic cells repopulate the satellite cell niche, thus maintaining and replenishing the quiescent satellite cell pool for subsequent rounds of regeneration (Collins et al., 2005; Shinin et al., 2006).

The role of satellite cells in postnatal growth has also been studied in limb muscles. In mice, the first 3 weeks of neonatal growth results in a threefold increase in muscle mass during which the satellite cell population undergoes a significant reduction from ~30% of myonuclei per myofiber down to 5%, following fusion with neonatal muscles. Parallel increases in myonuclear numbers and cytoplasmic proteins occur up to postnatal day 21 (White et al., 2010). After postnatal day 21, satellite cells enter into a quiescent cellular state under the regulation of Notch signaling (Fukada et al., 2011), but myofiber size continues to increase without the addition of new myonuclei (White et al., 2010). Recent satellite cell ablation studies have also shown that myonuclear addition from satellite cells is dispensable for hypertrophic growth of limb muscles in the adult (McCarthy et al., 2011). Furthermore, satellite cells do not appear to be required for maintenance of most adult limb muscles. A recent satellite cell ablation study found that loss of >90% of adult limb satellite cells failed to alter muscle size or myofiber type in five different limb muscles with aging (Fry et al., 2015). However, myonuclear addition does occur at a basal level in uninjured postnatal limb muscles and may be required for maintenance of extensor digitorum longus myofiber size with aging (Keefe et al., 2015). Together, these studies suggest that the initial phase of postnatal muscle growth occurs with the addition of myonuclei from satellite cells, but maintenance of most adult limb muscle size is not dependent on satellite cells.

Regulatory genes involved in satellite cell biology have also been elucidated from studies of limb muscle. In adult skeletal muscle, quiescent satellite cells express Pax7, a transcription factor that specifies the myogenic lineage (Seale et al., 2000). Once activated, satellite cells exit cellular quiescence, enter the cell cycle, and begin progression through the myogenic lineage under the control of myogenic regulatory factors (MRFs), muscle-specific transcription factors of the basic-helix-loop-helix (bHLH) class, including myogenic differentiation protein (MyoD), myogenic factor 5 (Myf5), myogenic regulatory factor 4 (Mrf4), and myogenin (Weintraub et al., 1991; Olson and Klein, 1994; Chang and Rudnicki, 2014). MyoD and Myf5 are expressed during the proliferative phase and regulate myogenic differentiation (Cooper et al., 1999; Valdez et al., 2000), while Mrf4 and myogenin are expressed upon terminal differentiation and exit from the cell cycle (Chang and Rudnicki, 2014).

Increasing evidence suggests that satellite cells within a muscle are heterogeneous (Motohashi and Asakura, 2014). Satellite cells containing high levels of Pax7 demonstrate slower proliferation rates, lower metabolism, and resistance toward differentiation, indicating a more “stem-like” phenotype compared to satellite cells with lower levels of Pax7 (Rocheteau et al., 2012). Various groups have also discovered distinct satellite cell subpopulations based on differential expression of other proteins including α7-integrin, β1-integrin, c-met, CD34, calcitonin receptor, C-X-C chemokine receptor type 4 (CXCR4), M-cadherin, Myf5, neural cell adhesion molecule 1, syndecans 3 and 4, and vascular cell adhesion molecule 1 (Rosen et al., 1992; Cornelison and Wold, 1997; Beauchamp et al., 2000; Blanco-Bose et al., 2001; Cornelison et al., 2001; Tamaki et al., 2002; Sherwood et al., 2004; Fukada et al., 2007; Ikemoto et al., 2007; Kuang et al., 2007; Kafadar et al., 2009). While the mechanisms underlying satellite cell heterogeneity are still being elucidated, growing evidence suggests that satellite cell biology is also variable in a muscle-dependent manner, as discussed below.

## The ‘Other’ Somite-derived Muscles: Epaxial, Diaphragm, Internal Larynx, and Tongue Satellite Cells

### Epaxial Satellite Cells

Epaxial skeletal muscles include the deep muscles of the back. Similar to hypaxial muscle development, epaxial muscles arise from the dorsomedial dermomyotome of the segmented paraxial mesoderm (**Figure 1**; Christ and Ordahl, 1995; Burke and Nowicki, 2003). While the development of epaxial muscle is well studied (Munsterberg et al., 1995; Tajbakhsh et al., 1997; Borycki et al., 1999; Gustafsson et al., 2002; McDermott et al., 2005; Borello et al., 2006; L’Honore et al., 2010; Sato et al., 2010), the biological properties of adult epaxial satellite cells remain largely unknown. Mouse models expressing *nLacZ* under the control of the *Pax3* promoter showed coexpression of Pax3 and Pax7 is retained in the majority of adult satellite cells of the deep ventral trunk muscles (Relaix et al., 2006). Pax3 expression was also maintained in cultured epaxial myoblasts, suggesting that Pax3 may contribute to both the quiescent and activated states of epaxial satellite cells (Relaix et al., 2006). How Pax3 contributes to adult epaxial satellite cell biology and the role of Pax3 in epaxial muscle regeneration remains to be determined, as well of other fundamental aspects of epaxial satellite cell biology and myogenesis.

### Diaphragm Satellite Cells

The diaphragm muscle is composed of three distinct domains: crural muscle, costal muscle, and a central tendonous domain (Anraku and Shargall, 2009). Recent studies provide evidence that the muscle components arise from the lateral dermomyotome of the cervical somites while the central tendonous connective tissue arises from the pleuroperitoneal folds of lateral plate origin (**Figure 1**; Noden et al., 1999; Mootoosamy and Dietrich, 2002; Pickering and Jones, 2002; Babiuk et al., 2003; Brown et al., 2005; Merrell et al., 2015). Postnatally, diaphragmatic satellite cells differ from hypaxial muscle in that Pax3 expression is maintained along with Pax7 and Myf5 (Relaix et al., 2006; Day et al., 2007; Stuelsatz et al., 2012). Recent studies indicate that satellite cell-derived myoblasts of the diaphragm behave differently than those of the hindlimb *in vitro*. Using the Nestin-GFP mouse model (Day et al., 2007) to identify self-renewal of myoblasts, Stuelsatz et al. (2014) found higher percentages of GFP^+^ cells in diaphragm verses limb muscle cultures. *In vitro* clonal expansion assays revealed that diaphragmatic myoblasts proliferated similarly to those of hindlimb muscles (Chen et al., 2011; Stuelsatz et al., 2012), but myogenic differentiation of diaphragm cultures consistently reached maximum fusion indexes earlier than limb cultures (Stuelsatz et al., 2014). However, other studies demonstrated that diaphragmatic satellite cells undergo increased proliferation and decreased differentiation *in vitro* when compared to limb, trunk, and craniofacial muscles (Ippolito et al., 2012). The discrepancies in these studies evidence the need for further examination of diaphragmatic satellite cell biology. *In vivo* studies also indicate some interesting biology associated with diaphragmatic satellite cells. *In vivo* lineage tracing studies examining the contribution of diaphragmatic satellite cells to uninjured diaphragm muscles revealed continued addition of new myonuclei with age, yet myofiber size was not affected with satellite cell ablation (Keefe et al., 2015). One study in rats suggests that heterogeneity exists in the response of diaphragmatic satellite cells to the growth promoting stimuli provided by hemi-diaphragm denervation. By examining satellite cell numbers associated with various fiber types, this study found that only type IIx/b myofibers of the compensating non-denervated hemi-diaphragm showed significant increases in satellite cell numbers in both juvenile and adult mice, while no changes in number were associated with Type I or IIa myofibers (Kawai et al., 2012). Taken together, our current understanding of diaphragmatic satellite cell biology is still rudimentary and warrants further study, both *in vivo* and *in vitro*.

### Intrinsic Laryngeal Satellite Cells

The intrinsic muscles of the larynx are critical for respiration, airway protection and vocalization and include the thyroarytenoid, posterior cricoarytenoid (PCA), and lateral cricoarytenoid muscles. The intrinsic laryngeal muscles arise from the occipital somites during development (**Figure 1**; Noden et al., 1999; Noden and Francis-West, 2006). In thyroarytenoid and PCA muscles, basal levels of myonuclear addition were discovered using BrdU lineage tracing, suggesting that uninjured laryngeal muscle contains a subset of proliferating satellite cells (Goding et al., 2005). *In vitro* studies also found increased proliferation rates associated with Erk1/2 phosphorylation in primary laryngeal muscle cultures compared to hindlimb cultures (Walz et al., 2008). In response to laryngeal denervation, laryngeal satellite cell activation and proliferation occurs *in vivo* within days following denervation (Kumai et al., 2007) with myonuclear addition occurring in all intrinsic laryngeal muscles analyzed (Shinners et al., 2006). Surprisingly, myogenic cells expressing MyoD and myogenin are still present in thyroarytenoid muscles 2 years post-denervation in humans, suggesting a unique prolonged regenerative potential in laryngeal muscle (Donghui et al., 2010). In spite of this, age-related decreases in laryngeal satellite cell density and impaired regeneration of thyroarytenoid muscles occur (Malmgren et al., 2000; Lee et al., 2012). Together, these studies provide intriguing insights into intrinsic laryngeal satellite cells, but further studies are needed to elucidate the molecular and regulatory mechanisms underlying their distinct biology.

### Tongue Satellite Cells

Tongue muscles arise from mixed mesodermal origins. The intrinsic muscles arise from the first occipital somite, while cranial paraxial mesoderm contributes to the formation of the exterior tongue muscles (**Figure 1**; Shuler and Dalrymple, 2001; Czajkowski et al., 2014). To date, knowledge of adult tongue muscle satellite cell biology is severely limited. A denervation study of the tongue muscle using ^3^H-thymidine to label proliferating cells revealed increased numbers of ^3^H-thymidine labeled myonuclei, which suggests fusion of proliferating satellite cells to the myofibers (McGeachie and Allbrook, 1978). Tongue-derived myoblasts have been isolated, cultured and successfully differentiated into nascent myotubes *in vitro*, but the myogenic characteristics of these cells are yet to be directly studied (Ternaux and Portalier, 1993; LaFramboise et al., 2003).

## Craniofacial Satellite Cells: Extraocular, Masseter, and Pharyngeal

### Extraocular Satellite Cells

Extraocular muscles (EOMs) are responsible for rotation and movement of the eye and include the superior oblique, inferior oblique, superior rectus, inferior rectus, lateral rectus, and medial rectus muscles. EOM and their associated satellite cells differ from other skeletal muscles in that they arise from the prechordal and cranial paraxial mesoderm of the first pharyngeal arch during development (**Figure 1**; Couly et al., 1992; Noden and Francis-West, 2006). Early studies examining the effects of aging and dystrophic disease suggested EOM muscles are distinct from their hindlimb counterparts. Aging studies suggested that EOMs were preferentially spared with aging (Porter et al., 1998; Schoser and Pongratz, 2006), while studies examining EOM involvement in Duchenne, Becker, and some limb girdle MDs also showed a preferential sparing of these muscles (Kaminski et al., 1992; Emery, 2002). In addition, satellite cells of EOM have unique gene expression profiles in comparison to quiescent satellite cells of hindlimb muscles (Porter and Baker, 1996; Pacheco-Pinedo et al., 2009). EOM satellite cells also demonstrate distinct biological differences when compared to hindlimb satellite cells. In several species, EOM satellite cells chronically proliferate *in vivo* (McLoon and Wirtschafter, 2002a, 2003; Wirtschafter et al., 2004; Christiansen and McLoon, 2006), which may in part be due to a specific highly proliferative subpopulation (Kallestad et al., 2011). The transcription factor *Pitx2* is expressed in postnatal EOM myogenic precursor cells that are of a CD34^+^/Sca1^-^/CD31^-^/CD45^-^ lineage, which is thought to contribute to the proliferative properties of EOM satellite cells (Hebert et al., 2013). In addition, aged EOM satellite cells maintain proliferative and self-renewal abilities out to 24 months of age *in vitro* (Stuelsatz et al., 2014). Furthermore, global and orbital EOM satellite cells contribute new myonuclei to EOM myofibers in the absence of injury (McLoon and Wirtschafter, 2002a,b, 2003; Wirtschafter et al., 2004; Keefe et al., 2015). Together, these studies highlight satellite cell biology that is distinct from hindlimb satellite cells. Because of their proliferative and self-renewal propensities, EOM satellite cells have been proposed to be ideal candidates for use in cell-based therapies of myopathic disease (McLoon et al., 2007; Kallestad et al., 2011; Stuelsatz et al., 2014). However, in transplantation experiments in which EOM satellite cells were injected into the TA muscle of the hindlimb, EOM satellite cells successfully engrafted into the novel niche, but lost EOM-specific phenotypes such as expression of Myh13 and slow-tonic myosin (Sambasivan et al., 2009). These data suggested that the phenotypes of EOM satellite cells may be controlled by the niche. In support of this hypothesis, a recent study suggested that PW1/peg^+^ interstitial cells (PICs), which are present in higher numbers in EOM compared to the TA, provide a promyogenic environment that contributes to the resistance of both EOM satellite cells and myofibers to dystrophic and age-related disease (Formicola et al., 2014). Together, these data raise some intriguing questions. What roles do intrinsic and extrinsic mechanisms have on EOM satellite cell biology? Can such mechanisms be manipulated to improve the quality of life for individuals suffering from myopathic diseases? Studies are still needed to further elucidate the mechanisms contributing to the unique phenotypes of these satellite cells.

### Masseter Satellite Cells

Adult masseter satellite cells arise from the first and second pharyngeal arches of the cranial paraxial mesoderm with contributions from the splanchnic mesoderm and express a unique transcription profile compared to hindlimb satellite cells (**Figure 1**; Kelly et al., 2004; Noden and Francis-West, 2006; Nathan et al., 2008; Sambasivan et al., 2009). Early *in vivo* studies demonstrated an impaired regenerative ability in masseter muscles compared to hindlimb muscle that was associated with a lower number of satellite cells during regeneration (Pavlath et al., 1998). Masseter satellite cells undergo prolonged periods of proliferation *in vitro* with a concurrent delay of differentiation onset (Ono et al., 2010), which potentially may contribute to the impaired regenerative response observed in acute injury to masseter muscle. In contrast, masseter satellite cells increase in number per myofiber with age while their proliferative capabilities decline *ex vivo* (Ono et al., 2010). What molecular pathways are involved with the age-associated increase in satellite cell numbers in masseter muscles verses the decrease in satellite cell numbers in other skeletal muscles? Little is known regarding the underlying molecular mechanisms driving the phenotypes of masseter satellite cells.

### Pharyngeal Satellite Cells

Swallowing depends on the synchronous contraction of seven major muscles lining the nasal, oral, and laryngeal pharynxes to ensure propulsion of food and liquid from the oral cavity into the esophagus (Donner et al., 1985; Rubesin et al., 1987; Ekberg et al., 2009). Pharyngeal muscles arise from the cranial paraxial mesoderm of the third and fourth pharyngeal arches with contributions from splanchnic mesoderm (**Figure 1**; Kelly et al., 2004; Noden and Francis-West, 2006; Nathan et al., 2008). Pharyngeal muscles include the stylopharyngeus, palatopharyngeus, salpingopharyngeus, and the superior, middle, and inferior pharyngeal constrictor muscles (Dutta and Basmajian, 1960; Himmelreich, 1973; Donner et al., 1985; Rubesin et al., 1987; Ekberg et al., 2009). The inferior pharyngeal constrictor can be subdivided into the cricopharyngeus and the thyropharyngeus muscles (Donner et al., 1985; Rubesin et al., 1987; Ekberg et al., 2009). Recent *in vivo* and *in vitro* studies indicate that pharyngeal satellite cells have unique biological characteristics compared to hindlimb satellite cells. Pharyngeal muscles contain increased numbers of activated and proliferating satellite cells and contribute new myonuclei to pharyngeal myofibers in the absence of induced injury (Randolph et al., 2015). Additionally, *in vitro* clonal assays suggest a highly proliferative subpopulation of pharyngeal satellite cells could be contributing to the proliferative phenotype (Randolph et al., 2015). *In vivo* ablation studies indicated that satellite cells were required to maintain myonuclear numbers in certain pharyngeal muscles under basal conditions, suggesting that pharyngeal muscles undergo myonuclear turnover and require myonuclear addition from ongoing satellite cell myogenesis (Randolph et al., 2015). Could pathologic mutations enhance myonuclear turnover in pharyngeal muscles to such an extent that pharyngeal satellite cells could no longer adequately supply enough myonuclei to maintain homeostasis? Do disease-causing mutations impair the basal myogenic phenotype of pharyngeal satellite cells, potentially contributing to the pathological sensitivity of pharyngeal muscles observed in some MDs? These questions and many more remain to be addressed. However, several studies have examined pharyngeal satellite cell biology in the context of oculopharyngeal muscular dystrophy and will be discussed below.

## Could Variable Satellite Cell Biology Contribute to the Differential Susceptibility of Certain Muscles to Dystrophic Mutations?

Muscular dystrophies are a group of degenerative muscle diseases due to mutations in proteins ranging in function such as sarcolemmal structure (Hoffman et al., 1987), nuclear envelope structure (Bione et al., 1994; Bonne et al., 1999; Mittelbronn et al., 2008), post-translational glycosylation (Bonnemann et al., 2014), and RNA binding (Brais et al., 1998; Kühn et al., 2009). Many mutations have been characterized in both skeletal muscle-specific and ubiquitously expressed genes, yet both manifest in muscular pathology. Intriguingly, each dystrophy affects a specific subset of skeletal muscles within the human body (Emery, 2002), suggesting that biological differences exist between individual muscles that predispose them to specific pathological etiologies.

Satellite cells have been implicated in the pathology of some MDs and may contribute to the variable muscle sensitivity observed in some dystrophies through several mechanisms (**Table 1**). For example, in response to chronic myofiber degeneration, satellite cells are subjected to multiple rounds of regeneration, which can “exhaust” the regenerative abilities of the satellite cell population over time (Webster and Blau, 1990; Decary et al., 2000; Morgan and Zammit, 2010; Sacco et al., 2010). Additionally, satellite cell impairment may occur early in the disease process if satellite cells express the mutant genes. Below we discuss the functional deficits in satellite cells observed in Duchenne MD (Blau et al., 1983; Webster and Blau, 1990; Sacco et al., 2010), Emery–Dreifuss MD (Favreau et al., 2004; Frock et al., 2006), facioscapulohumeral MD (Winokur et al., 2003; Barro et al., 2010), myotonic dystrophy (Furling et al., 2001; Thornell et al., 2009), oculopharyngeal MD (Périé et al., 2006), and some congenital MDs as well (Castets et al., 2011; Urciuolo et al., 2013).

### Duchenne Muscular Dystrophy

Duchenne muscular dystrophy (DMD) is an early onset childhood X-linked disease associated with the absence of dystrophin (Hoffman et al., 1987), a sarcolemma-associated cytoplasmic protein critical for maintaining sarcolemmal integrity of myofibers (Durbeej and Campbell, 2002). Minimal levels of mechanical stress are needed to impair sarcolemmal integrity in the absence of dystrophin, resulting in recurrent rounds of myofiber damage and repair (Petrof et al., 1993). Patients suffering from DMD experience progressive loss of muscle function, eventually leading to death before the age of 30. The main skeletal muscles affected in DMD are found in the shoulder, upper limbs, hips, thighs, and calves (Emery, 2002). Life-threatening symptoms for many patients involve cardiac and respiratory failure from impairment of the heart and diaphragm muscles, respectively (Nigro et al., 1990; Stedman et al., 1991). Of note, craniofacial muscles, such as the extraocular and internal laryngeal muscles, are mostly spared in DMD with the exception of pharyngeal muscles in advanced stages of the disease (Kaminski et al., 1992; Emery, 2002; Marques et al., 2007; Shinonaga et al., 2008). The mechanism of resistance of EOM to dystrophic changes was recently addressed in irradiation studies using *mdx:utrophin* heterozygous mice. EOMs of these mice failed to develop dystrophic phenotypes even after receiving 18 Gy gamma irradiation. This resistance to dystrophic change was attributed to the presence of multiple EOM myogenic precursor populations that prevented loss of myofiber size, suggesting that the proliferative satellite cell populations of EOM play a role in the muscle sparing of EOM in many dystrophies (McDonald et al., 2014). Satellite cell abnormalities are noted in dystrophin-deficient hindlimb muscles of both mice and humans at early stages of disease. In mouse hindlimb muscles, impaired satellite cell attachment to *mdx* myofibers may contribute to the pre-myonecrosis myofiber hypotrophy found in early postnatal disease (Duddy et al., 2015). Furthermore, premature cell senescence is observed in cultured myoblasts isolated from limb muscles of DMD patients as early as 2 years of age, the age of typical clinical onset (Blau et al., 1983; Webster and Blau, 1990) attributed to both deficiencies in Notch signaling and telomeric shortening following repeated regenerative cycles (Mouly et al., 2005; Sacco et al., 2010; Jiang et al., 2014).

### Limb-Girdle Muscular Dystrophy

Limb-girdle muscular dystrophies (LGMD) are associated with mutations of more than 20 different genes in both muscle-specific and ubiquitously expressed genes with a range of molecular functions (Vieira et al., 2014). These include sarcomere proteins (titin), sarcolemmal proteins (sarcoglycan), glycosyltransferases (fukutin), nuclear envelope proteins (lamin A/C), and RNA-processing proteins (HNRPDL), to name a few. Intriguingly, despite the vast etiological variation, all mutations elicit dystrophic changes in muscles of the upper limb, shoulder, chest, hip, and upper leg (Broglio et al., 2010; Mitsuhashi and Kang, 2012). Satellite cell involvement has been implicated in some LGMDs. Biopsies from LGMD2A patients demonstrated a decrease in miR-1 and miR206, microRNAs involved in facilitating satellite cell differentiation that correlated with an increased Pax7+ population. Despite the increased Pax7+ population, regeneration was impaired and fibrosis elevated, suggesting an impairment of satellite cell transition from proliferation to differentiation could be contributing to the pathology of LGMD2A (Rosales et al., 2013). In contrast, decreased satellite cell numbers were reported in patients with α-, β-, or γ-sarcoglycan mutations (LGMD2D, 2E, and 2C, respectively) when compared to Becker muscular dystrophy patient samples (Higuchi et al., 1999). Additionally, murine POMGnT1-null myoblasts (representative of LGMD2O) demonstrated impaired proliferation *in vitro* (Yoshida et al., 2001; Miyagoe-Suzuki et al., 2009). In a mouse model for LGMD2H, knockout of E3 ubiquitin ligase tripartite motif-containing 32 (TRIM32) resulted in satellite cell senescence both *in vitro* and *in vivo* (Kudryashova et al., 2012; Mokhonova et al., 2015). Interestingly, pharmacologic induction of follistatin expression in satellite cells using the deacetylase inhibitor, trichostatin A, has proved beneficial in restoring myofiber size in α-sarcoglycan-deficient LGMD *in vivo* (Minetti et al., 2006). Of note, *in vitro* treatment of α-sarcoglycan-deficient murine satellite cells with trichostatin A resulted in hypernucleated myotubes, suggesting a pharmacologic enhancement of myoblast differentiation/fusion (Minetti et al., 2006). It remains to be seen if other pharmacolgic approaches that alter satellite cell function might also benefit LGMD patients with other mutations.

### Emery–Dreifuss Muscular Dystrophy

Emery–Dreifuss muscular dystrophy results in progressive weakness of the shoulder, upper limb, and calf muscles of patients. The most common forms of this dystrophy are caused by mutations in the ubiquitously expressed nuclear envelope proteins emerin, lamin A, or lamin C (Helbling-Leclerc et al., 2002). Why skeletal and cardiac muscles are preferentially affected in this disease, is still unclear. Patients with Emery–Dreifuss MD can have severe cardiac pathology occurring as early as 30 years of age (Vohanka et al., 2001; Emery, 2002; Broglio et al., 2010). *In vitro* studies using primary muscle cultures from *Lmna^-/-^* knockout mice, overexpression of mutant lamin A^R453W^, or RNAi knockdown of *emerin* demonstrated defects in myoblast differentiation (Favreau et al., 2004; Frock et al., 2006). In a recent study, *in vitro* culture of patient-derived myoblasts lacking emerin demonstrated enhanced proliferation with spontaneous differentiation, compared to control myoblasts, thus suggesting that satellite cell impairment could play a role in Emery–Dreifuss MD (Meinke et al., 2015).

### Facioscapulohumeral Muscular Dystrophy

Facioscapulohumeral muscular dystrophy (FSHD) is named for the muscles mainly affected in the disease, facial, shoulder, and upper arm muscles, but foot and pelvic-girdle muscles can also be affected (Tawil and Van Der Maarel, 2006). Of the dystrophies affecting craniofacial muscles, FSHD carries the best prognosis for long-term survival, as it is a slowly progressive disease that rarely affects the heart or the ability to breathe (Tawil and Van Der Maarel, 2006). The causative deletion for FSHD type 1 (FSHD1) occurs in the subtelomeric region of chromosome 4, which can induce the expression of genes such as *FSHD region gene 1* (*FGR1*), *FGR2*, *ANT1*, *DUX4*, and *DUX4c* (Gabellini et al., 2002; Dixit et al., 2007; Ansseau et al., 2009; Bodega et al., 2009; Snider et al., 2010). The pathogenic contributions of these genes to FSHD are still being dissected. However, evidence for satellite cell involvement in FSHD1 is growing. *DUX4* expression in cultured myoblasts inhibited myogenic differentiation by repression of *Myf5* and *MyoD* (Bosnakovski et al., 2008a,b, 2009), while overexpression of DUX4 was toxic to myoblasts *in vitro* (Kowaljow et al., 2007). In contrast, overexpression of DUX4c stimulated myoblast proliferation but inhibited differentiation *in vitro* (Bosnakovski et al., 2008a; Ansseau et al., 2009). Additionally, FGR1 overexpression impaired myoblast proliferation as well as myoblast fusion (Chen et al., 2011; Feeney et al., 2015). Primary myoblasts collected from affected thigh muscles of a transgenic mouse overexpressing FGR1 produced smaller clonal colonies than myoblasts derived from the unaffected diaphragm muscle (Chen et al., 2011). Taken together, these results indicate that satellite cells could play a direct role in FSHD1 pathology. The mechanism(s) underlying the muscle specificity of these altered myogenic phenotypes remains to be determined.

### Myotonic Dystrophy

Myotonic dystrophy (DM) is a complex, multisystemic group of dystrophies that genetically arise from untranslated repeat nucleotide expansions of two separate genes, *dystophia myotonic protein kinase* (*DMPK*) and *zinc finger protein 9* (*ZNF9*) (Day and Ranum, 2005). A (CTG)_80-4000_ repeat in the 3′ untranslated region of *DMPK* is present in patients with myotonic dystrophy type 1 (DM1). The expanded regions of *DMPK* transcripts result in altered RNA biogenesis and processing of multiple transcripts, in part, by the sequestration of the splicing factor muscle blind (MBNL1) and stabilization of CUG-binding protein 1 (CUGBP1; Mastroyiannopoulos et al., 2010). In myotonic dystrophy type 2 (DM2), up to 75–11,000 repeat expansions of (TG)_n_(TCTG)_n_(CCTG)_n_ reside in intron 1 of *ZNF9* (Day and Ranum, 2005), dysregulating alternative slicing as well as protein production by sequestration of the 20S proteasome (Salisbury et al., 2009). While DM1 and DM2 result from distinct genetic mutations, the biological consequences are similar as myotonia, muscular dystrophy, muscle pain, cataracts, cardiac arrhythmias, insulin insensitivity, and diabetes, hypogammaglobulinemia, and testicular failure occur in both (Schoser and Timchenko, 2010). DM affects muscles of the eyelid, face, neck, lower arms, and legs, diaphragm, and intercostal muscles (Batten and Gibb, 1909; Zifko et al., 1996). However, DM1 is associated with muscle weakness and atrophy in the lower limb muscles, while in DM2 the disease is more predominant in the upper limbs (Tieleman et al., 2012). Life-threatening conditions involving cardiac disease, respiratory failure, and difficulties in swallowing can occur (Zifko et al., 1996; Tieleman et al., 2009, 2012). Satellite cell number, proliferation and differentiation are differentially altered in DM1 patients. Decreased satellite cell numbers may result from induced autophagic processes in DM1 myoblasts (Beffy et al., 2010). Cultured myoblasts, obtained from affected lower limb muscles, proliferated less compared to unaffected upper limb muscle cultures derived from the same patients (Thornell et al., 2009). Enhanced expression of prostaglandin E2 by DM1 myoblasts inhibited differentiation and fusion in an autocrine manner (Beaulieu et al., 2012). Additionally, the p16-pathway induced premature senescence in DM1 myoblasts (Bigot et al., 2009). Satellite cells have also been implicated in DM2 pathology. For example, satellite cells underwent premature senescence in DM2 patients, but in a non-p16 dependent manner through a telomere-driven pathway (Malatesta et al., 2011; Renna et al., 2014). Impairment of satellite cells could be a major pathologic determinant in myotonic dystrophies.

### Oculopharyngeal Muscular Dystrophy

Oculopharyngeal muscular dystrophy (OPMD) is an autosomal dominant disease, which typically affects people older than 50 years of age (Abu-Baker and Rouleau, 2007; Messaed and Rouleau, 2009). An aberrant expansion of alanines in the N-terminus of poly adenosine binding protein nuclear one (PABPN1) is the underlying cause of this presently incurable disease (Brais et al., 1998; Abu-Baker and Rouleau, 2007; Messaed and Rouleau, 2009). PABPN1 is a ubiquitously expressed protein that plays key roles in RNA biogenesis (Banerjee et al., 2013). The endogenous protein contains a 10-alanine repeat at its N-terminus, but expansions resulting in 12–18 alanines are reported in OPMD patients (Brais et al., 1998; Abu-Baker and Rouleau, 2007; Messaed and Rouleau, 2009; Jouan et al., 2014). Muscle weakness and dystrophy occur preferentially in craniofacial skeletal muscles including the upper eye-lid, pharynx, EOMs, and tongue with weakness in upper limb muscles developing later in the disease (Emery, 2002; Abu-Baker and Rouleau, 2007; Messaed and Rouleau, 2009).

The major life-threatening difficulty for OPMD patients is the resultant dysphagia, or impairments in swallowing (Périé et al., 2006). Pharyngeal muscles of the nasal, oral, and laryngeal pharynxes are essential components of the swallow reflex (Miller, 2002; Ertekin and Aydogdu, 2003; Miller, 2008), which prevents aspiration of food and water into the trachea and lungs and the formation of life-threatening pneumonia (Martin et al., 1994; Prasse and Kikano, 2009). Of note, decreased proliferation of pharyngeal satellite cells isolated from OPMD patients was observed *in vitro* (Périé et al., 2006). In transgenic mice that overexpress wild-type *PABPN1* specifically in skeletal muscle, increased numbers of myofibers with central nuclei suggested a positive effect of wild-type PABPN1 on satellite cell fusion *in vivo* (Randolph et al., 2014). Together, the above studies suggest that PABPN1 plays a critical role in pharyngeal satellite cell myogenesis and mutations in this protein may contribute to satellite cell impairment in OPMD patients.

Results from recent clinical trials provide preliminary evidence for the use of satellite cell transplantation as a therapeutic treatment for dysphagic OPMD patients. Phase I/IIa clinical trials were performed with dysphagic OPMD patients in which myoblasts obtained from unaffected skeletal muscles were amplified in culture and transplanted into cricopharyngeal muscles following surgical treatment of the cricopharyngeal muscle. Patients receiving injections of larger numbers of myoblasts into the cricopharyngeal area demonstrated significant improvement in swallowing over a 2-years period (Perie et al., 2014), thus providing experimental support for the use of satellite cell-based therapies for OPMD patients.

### Congenital Muscular Dystrophies

Congenital muscular dystrophies (CMD) represent a large group of congenital onset muscle diseases. While this group of muscle diseases has been widely studied, satellite cell involvement has only been implicated in the pathology of two forms to date: collagen VI-related myopathies (COL6-RD) and selenoprotein N-related myopathies (SEPN1-RM). COL6-RD include Ullrich MD and Bethlem myopathy and arise from mutations in COL6A1, COL6A2, or COL6A3 (Bonnemann et al., 2014). Muscle weakness occurs in distal limb, neck flexor muscles, lumbar, intercostals, and diaphragm, as well as proximal limb muscles such as the quadriceps, biceps and triceps (Haq et al., 1999; Camacho Vanegas et al., 2001; Quijano-Roy et al., 2012). When human biopsy samples were examined for changes in Pax7^+^ satellite cell numbers in normal verses Ullrich MD, no changes were observed (Paco et al., 2012). However, in *Col6a1* knockout mice, satellite cell self-renewal was impaired following multiple bouts of induced injury while satellite cell numbers were maintained in *Col6a1^-/-^* mice when treated with cyclosporin A (Urciuolo et al., 2013; Gattazzo et al., 2014). Whether these results are recapitulated in satellite cells from Ullrich MD patients has yet to be determined. Rigid spine syndrome related to *SEPN1* is a CMD within the SEPN1-related myopathies. Affected muscles include the thigh, gluteus maximus, paravertebral, intercostal, and sternocleidomastoid muscles (Quijano-Roy et al., 2012). Studies examining satellite cells in *Sepn1-/-* mice revealed decreased satellite cell numbers, impaired self-renewal, enhanced satellite cell proliferation, and exhaustion of the satellite cell pool following one round of regeneration (Castets et al., 2011). These studies suggest a potential role for satellite cells in the pathology of some CMDs. Further studies are needed to determine if satellite cells would be beneficial therapeutic targets for CMD patients.

## Summary

Skeletal muscles are a highly diverse and dynamic group of tissues. As discussed, many factors contribute to skeletal muscle diversity including embryologic origin, gene expression, and functional/metabolic requirements. Such diversity likely contributes to the pathologic sensitivities of different skeletal muscles to aging and disease. Unfortunately, little is known about the effects of age or disease on non-limb muscles as a whole or what factors predispose them to the effects of pathologic conditions. Additionally, satellite cells could serve as pathologic determinants in some dystrophies; however, our knowledge of non-limb satellite cells and their role in muscle biology is severely lacking. Recognizing and elucidating the distinct differences in satellite cell biology between different skeletal muscles could be the key to unraveling the conundrum of muscle specificity between the various MDs. This review highlights the potential benefit of exploring satellite cell biology of non-limb skeletal muscles for the development of novel therapeutic approaches for patients suffering from MDs.

## Author Contributions

MR: Conception and design, financial support, collection, and assembly of data, manuscript writing, final approval of manuscript. GP: Conception and design, financial support, administrative support, collection, and assembly of data, manuscript writing, final approval of manuscript.

## Conflict of Interest Statement

The authors declare that the research was conducted in the absence of any commercial or financial relationships that could be construed as a potential conflict of interest.
